# Photoreceptors’ gene expression of *Arabidopsis thaliana* grown with biophilic LED-sourced lighting systems

**DOI:** 10.1371/journal.pone.0269868

**Published:** 2022-06-10

**Authors:** Peter Beatrice, Donato Chiatante, Gabriella Stefania Scippa, Antonio Montagnoli

**Affiliations:** 1 Department of Biotechnology and Life Sciences, University of Insubria, Varese (VA), Italy; 2 Department of Biosciences and Territory, University of Molise, Pesche (IS), Italy; Karlsruhe Institute of Technology, GERMANY

## Abstract

Using specific photoreceptors, plants can sense light signals fundamental to their growth and development under changing light conditions. Phytochromes sense red and far-red light, cryptochromes and phototropins sense UV-A and blue light, while the *UVR8* gene senses UV-B signals. The study of the molecular mechanisms used by plants to respond to artificial biophilic lighting is of pivotal importance for the implementation of biophilic approaches in indoor environments. CoeLux® is a new lighting system that reproduces the effect of natural sunlight entering through an opening in the ceiling, with a realistic sun perceived at an infinite distance surrounded by a clear blue sky. We used the model plant *Arabidopsis thaliana* to assess the gene expression of the main plant photoreceptors at different light intensities and at different times after exposure to the CoeLux® light type, using high-pressure sodium (HPS) lamps as control light type. Genes belonging to different families of photoreceptors showed a similar expression pattern, suggesting the existence of a common upstream regulation of mRNA transcription. In particular, *PHYA*, *PHYC*, *PHYD*, *CRY1*, *CRY2*, *PHOT1*, and *UVR8*, showed a common expression pattern with marked differences between the two light types applied; under the HPS light type, the expression levels are raising with the decrease of light intensity, while under the CoeLux® light type, the expression levels remain nearly constant at a high fold. Moreover, we showed that under biophilic illumination the light spectrum plays a crucial role in the response of plants to light intensity, both at the molecular and morphological levels.

## Introduction

Plants are photo-autotrophic and sessile organisms dependent upon light for their survival [1]. To adapt to a changing light environment, plants constantly monitor the quantity, quality, and direction of incident light [2]. To achieve this, plants possess several photoreceptors proteins that perceive a broad light spectrum spanning from UV-B (280 nm) to far-red (750 nm). The light stimuli collected by photoreceptors will modify a multitude of cellular physiological processes, optimizing photosynthesis, minimizing photo-damage and influencing both the architecture and growth of the plant [3]. Three principal families of signal-transducing photoreceptors have been identified and characterized in higher plants’ tissues: phytochromes, cryptochromes, and phototropins [2]. Phytochromes (PHYs) were the first photosensory receptors discovered in plants. In *Arabidopsis thaliana*, this family comprises five genes (*PHYA*-*PHYE*) involved in the sensing of red (R) and far-red (FR) light, with different functions throughout the plant life cycle including germination, de-etiolation, roots development, stomata development, flowering, and shade avoidance responses [3]. In response to low R/FR ratio signals and low irradiance, many plants display a pronounced increase in the elongation growth rate of stems and petioles, often at the expense of leaves and roots development [4]. This response, termed shade-avoidance syndrome (SAS), serves to move leaves toward better light conditions and provides an essential survival strategy in situations of canopy closure and limiting light. The promotion of the SAS is achieved through the regulation of the equilibrium between the two forms of the PHYs proteins, the inactive Pr form and the biologically active Pfr form, that translocate in the nucleus and interact with phytochrome-interacting factors (PIFs) [5]. Cryptochromes (CRYs) are UV-A and blue (B) light photoreceptors involved in functions like hypocotyl elongation, de-etiolation, stem elongation, leaf expansion, root elongation, flowering, anthocyanin accumulation, and regulation of the circadian clock [6]. In *A*. *thaliana* three genes are currently known in this family, respectively *CRY1* to *CRY3*, however, the role of *CRY3* is still unknown. In the absence of light, CRYs are inactive in the form of monomers [7]. Light-dependent dimerization triggers the activation of CRYs that start to interact with CRY-signalling proteins, such as PIFs [8] and COP1 (constitutive photomorphogenic 1) [9]. Phototropins (PHOTs) are UV-A and blue light photoreceptors represented by two genes (*PHOT1* and *PHOT2*) with strongly overlapping functions in *A*. *thaliana*. Both photoreceptors are responsible for phototropism of shoot and root, in addition, they regulate leaf shape, stomatal opening, accumulation of chloroplasts, and lateral roots elongation [10]. When PHOTs perceive light, the receptor activates through conformational changes and autophosporylation, and starts to interact with COP1 and SUMO (small ubiquitin-related modifier) [11]. Furthermore, in *A*. *thaliana* UV-B signals can be perceived by the UV resistance locus 8 protein (*UVR8*). This photoreceptor inhibits shade avoidance, hypocotyl elongation, petiole elongation, and rosette expansion. Many of the phenotypic effects mediated by UVR8 are opposite to those induced by shade [12]. This photoreceptor has a homodimeric resting state that on absorbance of UV-B monomerises in an active form, allowing the interaction with downstream signalling proteins like COP1 [13]. At the morphological level, a reduction in the lamina to petiole length ratio (L/P) is considered a hallmark response in *A*. *thaliana* plants growing under unfavourable light [14] and can be used to monitor the development of *A*. *thaliana* plants under different light intensities and spectra [15]. This morphological response helps the plant to move its photosynthetic organs toward better light conditions, in an effort to collect more light and improve photosynthesis.

The study of the mechanisms used by plants to respond to artificially illuminated environments is of pivotal importance for the implementation of biophilic approaches in indoor environments. The biophilia hypothesis indicates that a shortage in human connection with nature can lead to a significant reduction in health, well-being, and performance. Numerous studies already demonstrated that introducing plants into offices can have significant positive effects on attention, creativity, and productivity perceived by the occupants [16], reducing anxiety and nervousness [17]. Furthermore, window views were demonstrated to further boost these positive effects [18]. In this context, the use of indoor plants in combination with the CoeLux® lighting system could provide a new approach to increase the quality of life in close environments where natural light is not available. CoeLux® is a new LED-sourced lighting system that reproduces the effect of natural sunlight entering through an opening in the ceiling, with a realistic sun perceived at an infinite distance surrounded by a clear blue sky [19, 20]. It has already been determined that this artificial skylight generates positive long-term psycho-physiological effects on humans, as well as the real counterpart [21]. However, the knowledge of how plants can grow and adapt to this light type is almost absent.

In our previous work [15], we characterized the intensity and spectra of the CoeLux® light type. Furthermore, we assessed the morphological and physiological responses of *A*. *thaliana* to this light type, observing a decrease in above and belowground biomass, a reduced L/P, and a lowered net photosynthetic rate. Coupling morpho-physiological traits with the expression of the main photoreceptors genes could improve the biophilic approach in close environments by increasing the knowledge about the molecular mechanisms underneath the responses of plants to the CoeLux® light type. This would allow to (i) identify genes that could provide a significant starting point for the development of CoeLux®-adapted plant strains, and thus (ii) gain a clear indication of the possibility to use this kind of lighting system for indoor plant growth. Since the plant response to light is related to the light-activated form of the photoreceptors and the following protein-level changes, we hypothesise that light signalling may also trigger an altered expression of the photoreceptors genes themselves, each gene responding peculiarly to the light characteristics. In particular, since the CoeLux® light type is characterized by high R/FR and low blue light intensity, we expect to observe (i) a higher expression of the genes of photoreceptors that sense blue light, like CRYs and PHOTs, (ii) a lower expression of the genes of photoreceptors that sense red light, i.e. PHYs, and (iii) a lower L/P in the loss-of-function mutant plants in respect to the WT plants.

To test our hypothesis, *A*. *thaliana* plants have undergone a long- and a short-light treatment in terms of time exposure to the CoeLux® light type, using high-pressure sodium (HPS) lamps as control light type. The gene expression of photoreceptors was assessed at different light intensities and at different times after the exposure to the CoeLux® light type.

## Materials and methods

### Plant material and growth conditions

**Fig 1:**
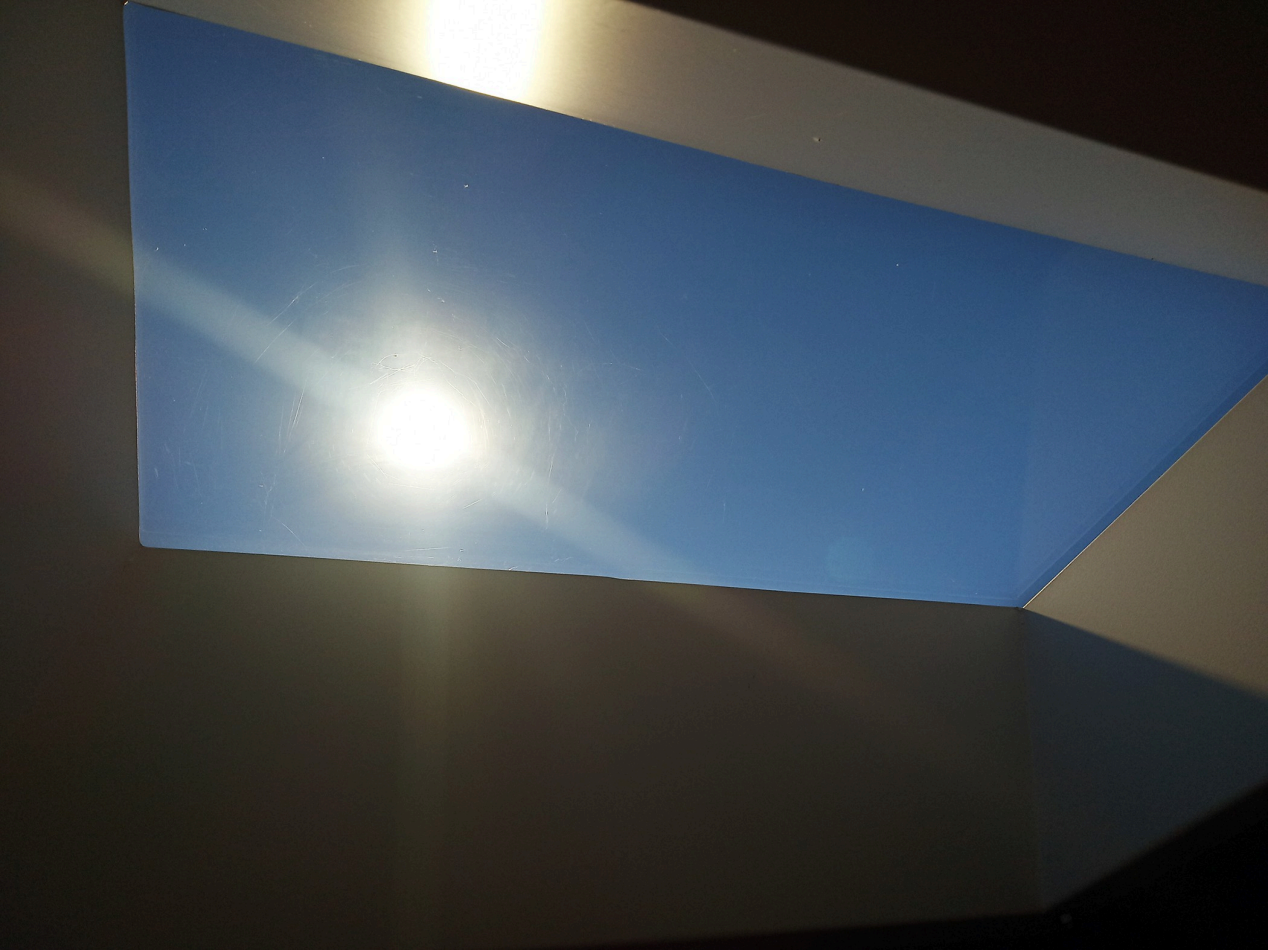
The visual appearance of the CoeLux® lighting system. The CoeLux® system is able to simulate the visual effect of the sun in a blue sky and project realistic shadows in the room, providing a real impression of natural sunlight together with all its properties.

*Arabidopsis thaliana* Col-8 wild-type (N60000) seeds and seeds of homozygous loss-of-function mutant lines *phyA* (N661576), *phyB* (N660754), *cry1* (N662234), and *cry2* (N3732) were purchased from the Eurasian Arabidopsis stock centre (NASC) [22]. The seeds were stratified at 4°C for 5 days on 1% agar gel and subsequently transferred to pot flats (Araflats; Arasystem; Ghent/Belgium) composed of 51 individual pot cavities with a 5 cm diameter, filled with sterilized commercial soil-less substrate. Plants were grown at a temperature as close as possible to 22°C, with an air humidity ranging between 50% and 70%, and a photoperiod of 14 h. Two different light sources were used: high-pressure sodium (HPS) lamps, considered as a standard light type in close environments plant production [23], and the LED-sourced CoeLux® systems, which are engineered to resemble the natural light and sun appearance (Fig 1).

**Fig 2:**
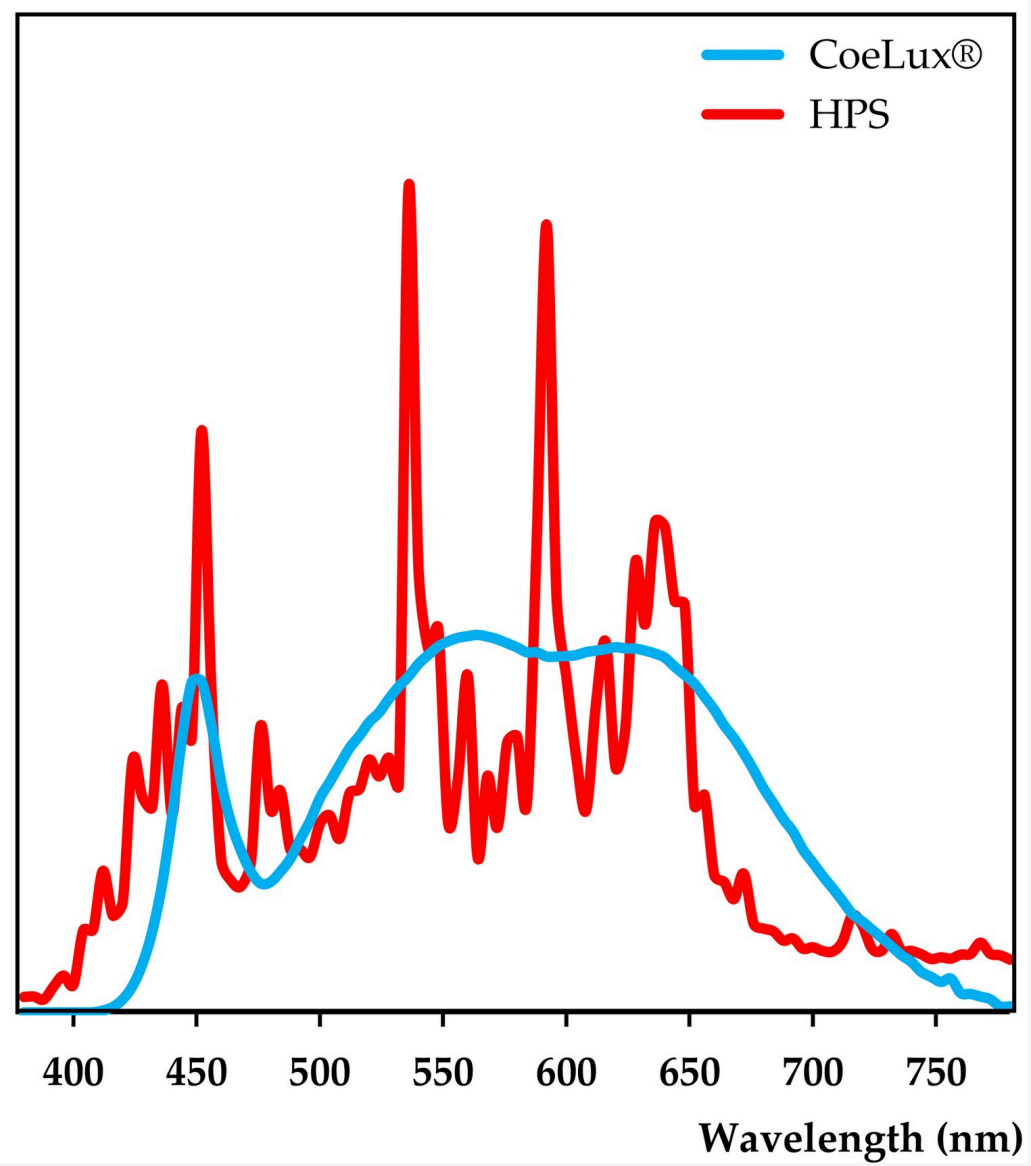
Mean spectra curves of the CoeLux® and HPS light types. Under both light types, at least 8 spectra measurements were collected in the range between 380 nm and 780 nm. To allow the comparison, the curves were normalised on the respective luminance value.

Both light types were characterized in a previous study [15], using the HD 2302.0 Light Meter (Delta Ohm) to measure the light intensity and the SpectraScan PR655 (Photo Research) to measure the spectra every 4 nm in the range between 380 nm and 780 nm (Fig 2). Briefly, the HPS light type has a higher blue component, while the CoeLux® light type has more yellow and red components (Table 1). Despite similar values of FR light, the red-to-far-red ratio (R/FR), calculated in the intervals (650–670 nm)/(720-740nm) [24], is higher under the CoeLux® light type (4.68) compared to the HPS light type (2.43). While the blue-to-green ratio (B/G), calculated in the intervals (420–490 nm)/(500-570nm) [24], is higher under the HPS light type (0.83) rather than under the CoeLux® light type (0.50).

**Table 1:** Spectra colour composition of the two light types used in this study.

Colour	Wavelength range (nm)	Relative intensity (%)	
		CoeLux®	HPS
BLUE	400–490	14	24
GREEN	490–560	24	24
YELLOW	560–590	15	11
RED	590–700	41	35
FAR-RED	700–780	6	6

For easier comparison, photon counts measurements were normalized on the luminance of the respective spectrum and the sum of normalized photon counts was calculated for the different wavelength intervals corresponding to the spectral colours [15]. Data are displayed in the form of relative intensity.

### Light treatments and experimental design

We subjected our plants to two different light treatments, respectively a Long-Term Light Treatment (LTLT) and a Short-Term Light Treatment (STLT). In the LTLT, plants were grown under the CoeLux® light type at progressive distances from the light source (20, 85, 205 cm), corresponding to decreasing values of light intensity (120, 70, 30 μmol m^-2^s^-1^). The fifth and sixth rosette leaves were sampled when the six-leaf stage was reached, between 17 and 30 days after sowing (DAS) depending on the light intensity, to analyse the gene expression in plants at the same phenological stage. Leaves from six different plants were sampled at 3 hours after dawn (HAD) and pooled together to obtain a single biological replica. Two biological replicas were collected and analysed independently. The leaves samples were rapidly harvested, flash-frozen in liquid nitrogen, and finely ground with pestle and mortar. The obtained powder was stored ad -80°C until RNA extraction. In the STLT, plants were grown under the HPS light type at 120 μmol m^-2^s^-1^ until the six-leaf stage was reached (17 DAS). Time 0 was sampled pre-dawn as described above. Subsequently, half of the plants were moved under the CoeLux® system at a distance of 20 cm (120 μmol m^-2^s^-1^) for the light treatment, while the other half remained under the HPS lamps as control light type. The fifth and sixth rosette leaves were sampled under both light types at 2, 6, 12, and 24 hours after both light sources were turned on. Leaves from six different plants were pooled together to obtain a single biological replica. At each sampling time, three biological replicates were collected and analysed independently. The loss-of-function mutant plants *phyA*, *phyB*, *cry1*, *cry2* and the respective WT controls were grown for 23 days at a light intensity of 120 μmol m^-2^s^-1^ under both light types.

### Lamina to petiole length ratio

In the case of the LTLT digital images of each plant were captured before sampling for the measurements of morphological traits. The lamina and petiole length of the fifth and sixth rosette leaves was measured using ImageJ (NIH, USA) and the lamina-to-petiole length ratio (L/P) was calculated. In the case of mutant plants, the whole rosette was sampled at 23 DAS and scanned at 800 dpi with the Epson Expression 12000XL instrument. The lamina and petiole length was measured on the two completely expanded younger leaves. To emphasize the different responses of mutant plants to the two light types, we normalized all L/P data on the mean value obtained from the WT plants grown under the respective light type.

### Gene expression analysis

Total RNA was extracted with the RNeasy Plant Mini Kit (Qiagen) according to the protocol of the manufacturer. RNA integrity was checked by electrophoresis on 1% agarose gel and RNA concentration was determined using a NanoDrop 2000 spectrophotometer (Thermo Scientific). The QuantiTect Reverse Transcription Kit (Qiagen) was used for the removal of genomic DNA contamination and the synthesis of cDNA. The NCBI Primer-BLAST tool was used to design primers pairs with an exon-exon junction span (Table 2).

**Table 2:** List of primers used in this study.

Gene	Locus	Primer sequence (5’ > 3’)	Source
*PHYA*	AT1G09570	TGAGCTGACTGGTCTTTCGG	This study
		CATTCTGCTCCTCAGTTCCTTCT	
*PHYB*	AT2G18790	CTCGTGCTTTGAGAGGGGAC	This study
		TCCAACAAAACAAACGCCGA	
*PHYC*	AT5G35840	TCCGCCATGAAGTGAAGGAC	This study
		TCCAGTTCCACATAGCCTTCTT	
*PHYD*	AT4G16250	AAGGCTCCAACAGGTTCTCG	This study
		CTGCACACGCCATTCTGAAC	
*PHYE*	AT4G18130	TGCAAAGCCCTACAAGGTGAA	This study
		TGACCAACGAAGCAGACACC	
*CRY1*	AT4G08920	GGGTTTCTAGGTGGTGGCTC	This study
		CGCACCAAAGACAATGGATCATA	
*CRY2*	AT1G04400	TGGACAATCCCGCGTTACAA	This study
		GCGTCCCATGGATGATGGAT	
*CRY3*	AT5G24850	GCATTCCCAAGCAAGCACAA	This study
		CTCTTTCGGAAGCCGACGTA	
*PHOT1*	AT3G45780	CACTGATCCTAGGCTTCCCG	Łabuz et al. [10]
		GTGGTTAGATCAGTCTCTGGACC	
*PHOT2*	AT5G58140	CCTGCACACCCCAGCTTATT	This study
		CTTGTATGACCAGCACCCGT	
*UVR8*	AT5G63860	TCAGGGAAAAGCTGGGTGTC	This study
		CATCCGTTAGGCCCGTTTCA	
*PP2AA3*	AT1G13320	TAACGTGGCCAAAATGATGC	Czechowski et al. [27]
		GTTCTCCACAACCGCTTGGT	
*UBQ10*	AT4G05320	GGCCTTGTATAATCCCTGATGAATAAG	Czechowski et al. [27]
		AAAGAGATAACAGGAACGGAAACATAGT	
*SAND*	AT2G28390	AGGATTGGGACCCCACAAGA	This study
		TATCGCCATCGCCTTGTCTG	

PHY: phytochrome; CRY: cryptochrome; PHOT: phototropin; UVR: ultraviolet receptor; PP2AA3: protein phosphatase 2A subunit A3; SAND: SAND family protein At2G28390; UBQ10: polyubiquitin 10.

Real-time PCR reactions were performed in a 20μL reaction mix composed of 10μL 2x SensiFAST SYBR No-Rox Mix (Meridian bioscience), 4.6μL H_2_O, 0.4μL primers mix (5 μM), and 5μL cDNA (2ng/μL). The amplification was carried out using the CFX Maestro thermocycler (Bio-Rad) under the following conditions: 95°C for 10 min, 40 cycles of 95°C for 15s and 60°C for 60s, then 95°C for 5s followed by the melting analysis. Three technical replicas were performed for each reaction. The geometrical mean [25] of three housekeeping genes (PP2AA3, UBQ10, and SAND) was used to normalize the gene expression levels via the 2^-ΔΔCT^ method [26]. In the LTLT, we set to 1 the expression levels measured in plants grown under the HPS light type at 120 μmol m^-2^s^-1^ (reference plants), while in the STLT, we set to 1 (reference line) the expression levels measured in plants sampled pre-dawn (time 0 in Fig 5).

Statistically significant differences (*p* < 0.05) between the two light treatments are marked with an asterisk and were calculated with SPSS Statistics 25 (IBM) via the Student’s t-test. Error bars represent the ±95% confidence interval (CI). In Fig 6, multiple comparisons were made with the post hoc Dunnett’s test. Statistically significant differences between the means (*p* < 0.05) were marked with the letters a, b, c for the HPS light type and with the letters x, y, z for the CoeLux® light type.

## Results

### Long-term light treatment

**Fig 3:**
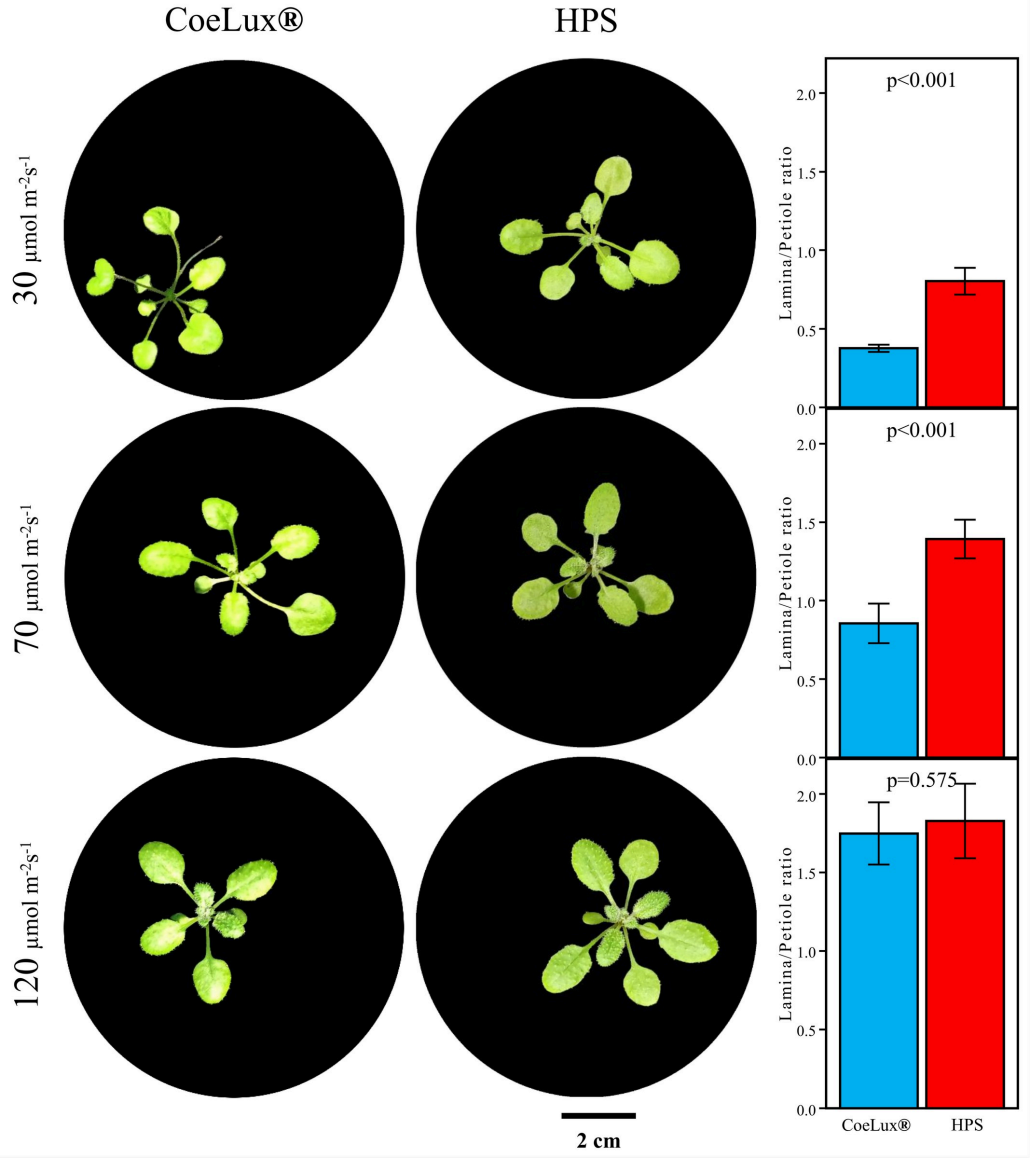
*A*. *thaliana* morphology in response to light treatments. Comparison of representative rosette phenotypes of plants at the 6-leaf phenological stage grown with constant light cycle (14:10) under the indicated light treatments. Data represent the means of n = 12 biological repeats ± 95% CI.

Three different light intensities were tested in the range between 30 and 120 μmol m^-2^s^-1^, corresponding to the light intensities that can be found inside the sunbeam of the CoeLux® lighting systems between 20 cm and 205 cm distance from the light source. Plants grown under lower light intensities showed a decreased L/P (Fig 3), suggesting the onset of a stronger shade avoidance syndrome (SAS). Furthermore, plants grown under the CoeLux® light type at 30 and 70 μmol m^-2^s^-1^ showed a significant lower L/P in respect to plants grown at the same light intensity under the HPS light type.

**Fig 4:**
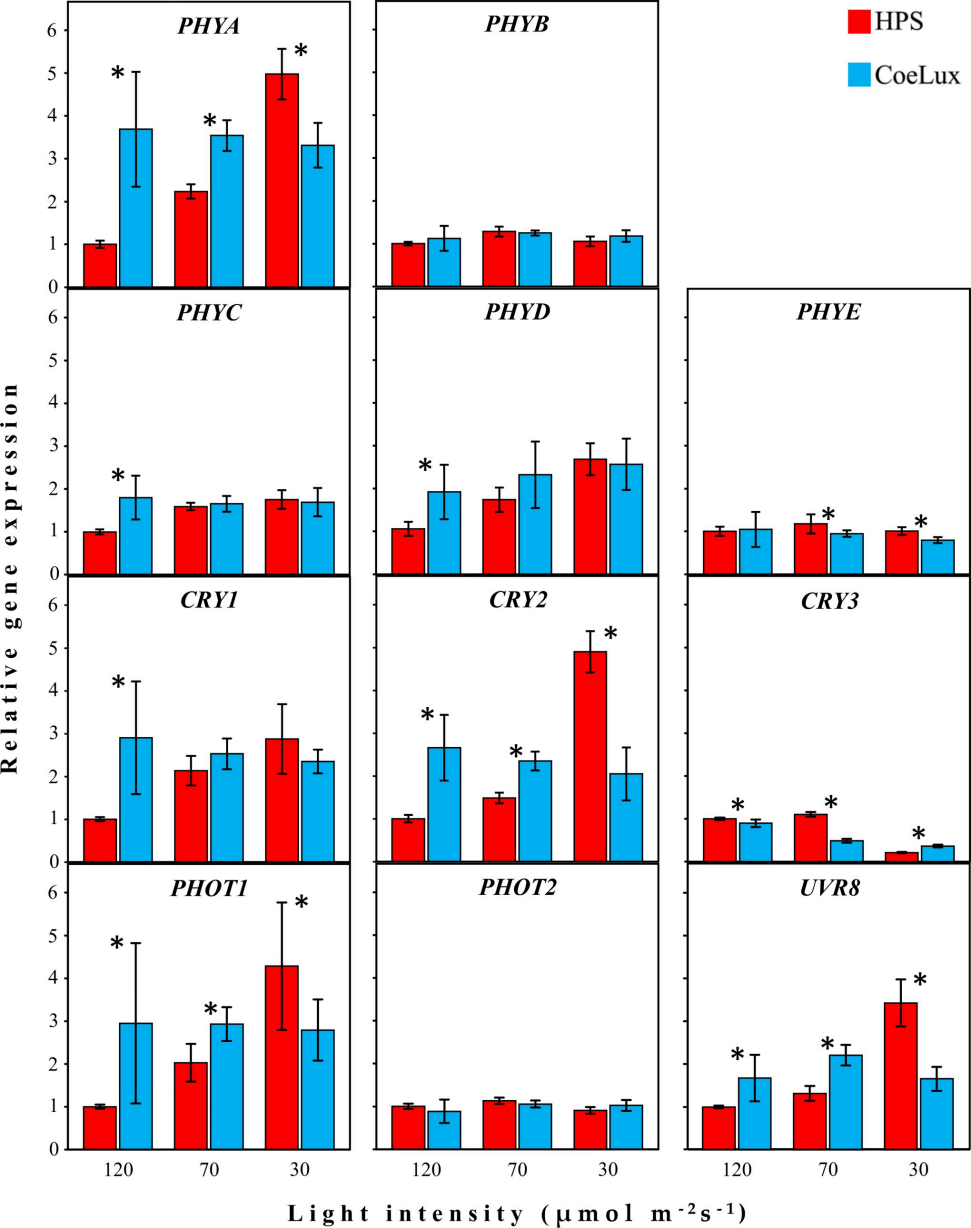
Relative expression of photoreceptors in the LTLT. The gene expression of the photoreceptors is relative to *A*. *thaliana* plants grown under the HPS light type at 120 μmol m^-2^s^-1^, measured in 6-stage rosette leaves of plants growing under constant light treatment. Data represent the means of n = 2 biological repeats ± 95% CI. Asterisks represent statistically significant differences (p < 0.05) between plants grown under the two light treatments.

The five PHYs genes of *A*. *thaliana* were analysed separately to identify all expression pattern variations among this family of photoreceptors. ***PHYA***—The *PHYA* gene showed a different expression pattern in plants grown under the two light types analysed (Fig 4 - *PHYA*). Under the HPS light type, the expression of the *PHYA* gene is raising with the decrease of the light intensity, reaching a 5-fold expression at 30 μmol m^-2^s^-1^. Under the CoeLux® light type, the expression of the *PHYA* gene is constant at all light intensities analysed, ranging between 3 and 4-fold the reference plants. A statistically significant difference between the two light types was observed at all light intensities analysed. ***PHYB***—The *PHYB* gene showed only minimal expression changes in response to the diverse light treatments and no significant differences were observed between the two light types analysed (Fig 4 - *PHYB*). ***PHYC***—The *PHYC* gene showed a different expression pattern in plants grown under the two light types analysed (Fig 4 - *PHYC*). Under the HPS light type, the expression of the *PHYC* gene is mildly raising with the decrease of the light intensity, reaching a 1.8-fold expression at 30 μmol m^-2^s^-1^. Under the CoeLux® light type, the expression of the *PHYC* gene is constant at all light intensities analysed, ranging around 1.7-fold the reference plants. A statistically significant difference between the two light types was observed at 120 μmol m^-2^s^-1^. ***PHYD***—The *PHYD* gene showed a similar expression pattern in plants grown under the two light types analysed (Fig 4 - *PHYD*). Under the HPS light type, the expression of the *PHYD* gene is raising with the decrease of the light intensity, reaching a 2.6-fold expression at 30 μmol m^-2^s^-1^. Under the CoeLux® light type, the *PHYD* gene showed a 1.8-fold expression at the higher light intensity and a mild expression increase with the decrease in the light intensity. A statistically significant difference between the two light types was observed at 120 μmol m^-2^s^-1^. ***PHYE***—The *PHYE* gene expression levels showed only small changes in response to both light spectrum and intensity. Under the HPS light type, the expression of the *PHYE* gene is constant at all light intensities analysed. Under the CoeLux® light type, the PHYE gene expression levels are decreasing with the decrease of the light intensity, reaching a 0.8-fold expression at 30 μmol m^-2^s^-1^. Statistically significant differences between the two light types were observed at 70 and 30 μmol m^-2^s^-1^.

The three CRYs genes of *A*. *thaliana* were analysed separately to identify all expression pattern variations among this family of photoreceptors. ***CRY1***—The *CRY1* gene showed a different expression pattern in plants grown under the two light types analysed (Fig 4 –*CRY1*). Under the HPS light type, the expression of the *CRY1* gene is raising with the decrease of the light intensity, reaching a 2.9-fold expression at 30 μmol m^-2^s^-1^. Under the CoeLux® light type, the expression of the *CRY1* gene is constant at all light intensities analysed, ranging between 2 and 3-fold the reference plants. A statistically significant difference between the two light types was observed at 120 μmol m^-2^s^-1^. ***CRY2***—The *CRY2* gene showed a different expression pattern in plants grown under the two light types analysed (Fig 4 –*CRY2*). Under the HPS light type, the expression of the *CRY2* gene is raising with the decrease of the light intensity, reaching a 4.9-fold expression at 30 μmol m^-2^s^-1^. Under the CoeLux® light type, the expression of the *CRY2* gene is constant at all light intensities analysed, ranging between 2 and 3-fold the reference plants. A statistically significant difference between the two light types was observed at all light intensities analysed. ***CRY3***—The *CRY3* gene showed a similar expression pattern in plants grown under the two light types analysed (Fig 4 –*CRY3*). Under the HPS light type, the expression of the *CRY3* gene showed no marked differences between 120 and 70 μmol m^-2^s^-1^, while a decrease down to 0.2-fold was observed at the lower light intensity analysed. Under the CoeLux® light type, the *CRY3* gene showed a decreasing expression with the decrease of the light intensity, also at 70 μmol m^-2^s^-1^, reaching a 0.4-fold expression at 30 μmol m^-2^s^-1^. A statistically significant difference between the two light types was observed at all light intensities analysed.

The two PHOTs genes of *A*. *thaliana* were analysed separately to identify all expression pattern variations among this small family of photoreceptors. ***PHOT1***—The *PHOT1* gene showed a different expression pattern in plants grown under the two light types analysed (Fig 4 - *PHOT1*). Under the HPS light type, the expression of the *PHOT1* gene is raising with the decrease of the light intensity, reaching a 4.3-fold expression at 30 μmol m^-2^s^-1^. Under the CoeLux® light type, the expression of the *PHOT1* gene is constant at all light intensities analysed, ranging between 2.8 and 2.9-fold the reference plants. A statistically significant difference between the two light types was observed at all light intensities analysed. ***PHOT2***—The *PHOT2* gene expression levels showed only minimal changes in response to both light spectrum and intensity, with no statistically significant difference between the two light types analysed (Fig 4 - *PHOT2*).

***UVR8***—The *UVR8* gene showed a different expression pattern in plants grown under the two light types analysed (Fig 4 - *UVR8*). Under the HPS light type, the expression of the *UVR8* gene is raising with the decrease of the light intensity, reaching a 3.4-fold expression at 30 μmol m^-2^s^-1^. Under the CoeLux® light type, the expression of the *UVR8* gene is constant at all light intensities analysed, ranging between 1.7 and 2.2-fold the reference plants. A statistically significant difference between the two light types was observed at all light intensities analysed.

### Short-term light treatment

In the STLT we worked at a constant light intensity to focus on short term gene activation or repression in response to the shift under the CoeLux® light type.

**Fig 5:**
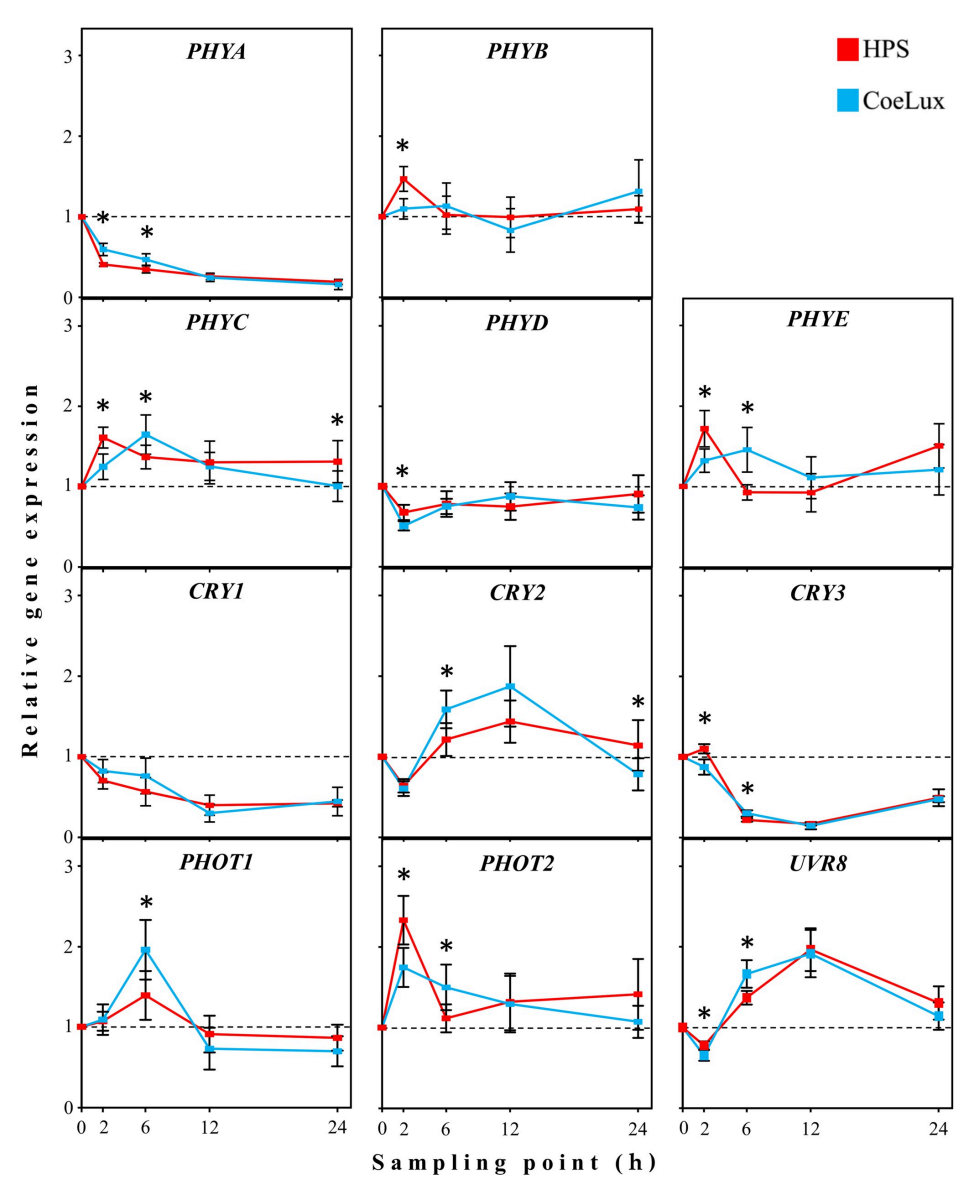
Relative expression of photoreceptors in the STLT. The gene expression of the photoreceptors in leaves of *A*. *thaliana* plants was measured after 2, 6, 12 and 24 h of light treatment and is shown as relative to the 0 h sampling point measured pre-dawn. Data represent the means of n = 3 biological repeats ± 95% CI. Asterisks represent statistically significant differences (p < 0.05) between plants grown under the two light treatments.

***PHYA***—Under the HPS light type, the *PHYA* gene showed a decreasing expression level with the proceeding of the lighting stimulation (Fig 5 - *PHYA*). The lower expression levels (0,2-fold the pre-dawn reference line) were measured at 24 hours after dawn (HAD). Under the CoeLux light type, the initial decrease was of lower magnitude and a statistically significant difference was observed at 2 and 6 HAD. ***PHYB***—Under the HPS light type, the *PHYB* gene showed an initial over-expression at 2 HAD followed by a return toward the pre-dawn reference line (Fig 5 - *PHYB*). Under the CoeLux light type, the *PHYB* gene showed no marked deviation from the reference line. A statistically significant difference between the two light types was observed only at 2 HAD. ***PHYC***- Under the HPS light type, the *PHYC* gene showed an initial over-expression (1.6-fold the reference line) at 2 HAD and a subsequent reduction to values tending to the pre-dawn reference line (Fig 5 - *PHYC*). Under the CoeLux light type, the response to the light turning on was slower, as the highest expression levels were measured at 6 HAD. A statistically significant difference between the two light types was observed at 2, 6, and 24 HAD. ***PHYD***—Under the HPS light type, the *PHYD* gene showed an initial down-expression followed by a return toward the pre-dawn reference line (Fig 5 - *PHYD*). Under the CoeLux light type, the initial down-expression was of higher magnitude, as a statistically significant difference between the two light types was observed at 2 HAD. ***PHYE***—Under the HPS light type, the expression of the *PHYE* gene showed an initial over-expression at 2 HAD, followed by a return toward the pre-dawn reference line at 6 and 12 HAD, and a subsequent over-expression at 24 HAD (Fig 5 - *PHYE*). Under the CoeLux light type, the *PHYE* gene showed a wider over-expression peak, ranging from 2 to 6 HAD, followed by a return toward the pre-dawn reference line. Statistically significant differences between the two light types were observed at 2 and 6 HAD.

***CRY1***—Under the HPS light type, the *CRY1* gene showed a decreasing expression level with the proceeding of the lighting treatment. The lower expression levels (0,4-fold the pre-dawn reference line) were measured at 12 and 24 HAD. Under the CoeLux light type, no statistically significant differences were observed compared to the HPS light type. ***CRY2***—Under the HPS light type, the *CRY2* gene showed an initial down-expression at 2 HAD followed by a wide up-regulation peak from 6 to 12 HAD and a subsequent return toward the pre-dawn reference line at 24 HAD (Fig 5 –*CRY2*). Under the CoeLux light type, the peak was of higher magnitude (up to 1.9-fold), and statistically significant differences between the two light types were observed at 6 and 24 HAD. ***CRY3***—Under the HPS light type, the *CRY3* gene showed an initial mild over-expression at 2 HAD followed by a decreasing expression level with the proceeding of the lighting stimulation (Fig 5 –*CRY3*). The lower expression levels (0,2-fold the pre-dawn reference line) were measured at 12 HAD. Under the CoeLux light type, no over-expression was observed at 2 HAD and a less pronounced decrease was observed at 6 HAD. Statistically significant differences between the two light types were observed at 2 and 6 HAD.

***PHOT1***—Under the HPS light type, the *PHOT1* gene showed an initial over-expression (1.4-fold the reference line) at 6 HAD and a subsequent reduction to values tending to the pre-dawn reference line (Fig 5 - *PHOT1*). Under the CoeLux light type, the initial over-expression was of higher magnitude (2-fold) and statistically different from the HPS light type. ***PHOT2***—Under the HPS light type, the *PHOT2* gene showed an initial over-expression (2.3-fold the reference line) at 2 HAD and a subsequent reduction to values tending to the pre-dawn reference line (Fig 5 - *PHOT2*). Under the CoeLux light type, the initial over-expression was of lower magnitude (1.7-fold) but extended till the 6 HAD sampling point. Statistically significant differences between the two light types were observed at 2 and 6 HAD.

***UVR8***—Under the HPS light type, the *UVR8* gene showed an initial down-expression at 2 HAD, followed by an up-regulation until 12 HAD and a return toward the pre-dawn reference line at 24 HAD (Fig 5 –*UVR8*). Under the CoeLux light type, the initial down-expression was of higher magnitude (0.6-fold the reference line), while higher expression levels were observed at 6 HAD. Statistically significant differences between the two light types were observed at 2 and 6 HAD.

### Mutant plants of photoreceptors genes

**Fig 6:**
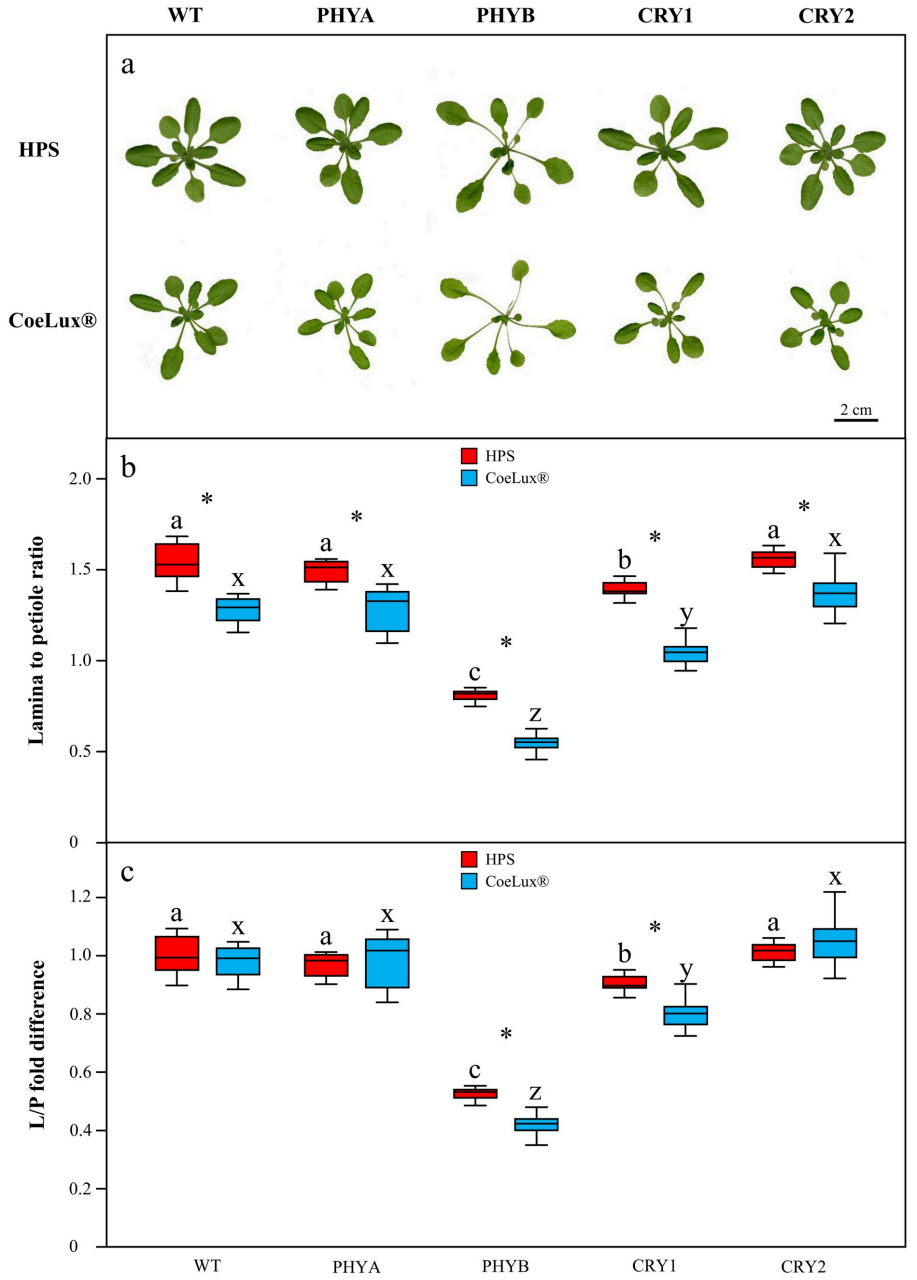
Morphology of mutants in response to the CoeLux® light type. (a) Comparison of representative rosette phenotypes of WT plants and mutant lines for the *PHYA*, *PHYB*, *CRY1*, and *CRY2* genes. (b) Lamina-to-petiole ratio of mutants grown under both light types, respectively in red under the HPS light type and blue under the CoeLux® light type. (c) Mutants data were normalised on the WT data mean to highlight mutant-specific responses. Vertical boxes represent approximately 50% of the observations (n = 12 biological repeats) and lines extending from each box are the upper and lower 25% of the distribution. Asterisks indicate statistically significant differences (*p* < 0.05) between plants grown under the CoeLux® and the HPS light type, while letters indicate differences (*p* < 0.05) between different mutants grown under the same light type.

As expected, plants grown under the CoeLux® light type showed a decreased L/P with respect to the control light type (Fig 6A and 6B). Compared to the WT, the mutants for the *PHYA* and *CRY2* genes showed no significant differences under both light types, while the mutants for the *PHYB* and *CRY1* genes showed a significantly lower L/P. In particular, the mutants for the *PHYB* gene showed a strong decrease in the L/P: 47% under the HPS light type and 58% under the CoeLux® light type. The normalisation of the mutants data on the respective WT mean, allows the identification of reductions of the L/P between the two light types stronger than that observed in the WT plants (Fig 6C), and, thus, specific responses of a selected genotype to the CoeLux® light type can be detected. The *PHYB* gene mutant showed a significant reduction from 0.53 fold to 0.42 fold, while the *CRY1* gene mutant showed a reduction from 0.89 fold to 0.80 fold.

## Discussion

Sensing light quality and intensity, photoreceptors play a key role in plant survival in changing light environments. The physiology and morphogenesis of plants are affected by the absolute and relative intensities of UV, blue, green, red and FR radiation. In particular, a reduced R/FR, an increased B/G, the low light intensity of the whole photosynthetically active radiation spectrum and low blue wavelengths are known to induce SAS responses [24, 28]. Compared to the HPS light type, the CoeLux® light type is characterized by a higher B/G and low blue light intensity. In our previous study, we identified these two light parameters as the main factors that could lead to the increased SAS observed in *A*. *thaliana* plants growing under the CoeLux® light type [15].

CRYs and PHOTs are the major photoreceptors families involved in the response to blue light attenuation [29, 30]. In particular, *CRY1* was reported to have a predominant role in the onset of the SAS in response to blue light attenuation caused by competition with other plants [14]. In the LTLT we observed a higher expression of *CRY1*, *CRY2* and *PHOT1* under the CoeLux® light type at 120 and 70 μmol m^-2^s^-1^, and a lower expression at 30 μmol m^-2^s^-1^ (Fig 4). However, under the CoeLux® light type, the L/P suggests the onset of a stronger SAS in plants grown at 30 and 70 μmol m^-2^s^-1^ (Fig 3). Thus, a higher or lower expression of CRYs or PHOTs can not be directly related to the onset of a stronger SAS at all light intensities. In the STLT these genes showed a diverse variety of responses over time, with no marked deviations in terms of higher or lower gene expression. The loss-of-function mutant of the *CRY2* gene responded as the WT to the CoeLux® light type, while the *cry1* mutant showed a more pronounced L/P decrease under this light type (Fig 6C), suggesting the involvement of this gene in the plants’ responses to the CoeLux® light type.

PHYs primarily sense red and FR light, but they were also reported to collaborate in the responses to blue light [31]. It is known that the phyA protein is rapidly degraded upon exposure to light [32]. Our STLT data suggest that light stimulation promotes the plan de-etiolation not only by phyA degradation but also by light-dependent under-expression of the *PHYA* gene. A higher expression of the *PHYA* gene could lead to the induction of a stronger SAS [28]. The slight delay in the *PHYA* switch off, observed in plants growing under the CoeLux® light type (Fig 5) could facilitate the onset of the more severe SAS observed under the CoeLux® light type. However, the loss of function of this gene has not led to a response to the CoeLux® light type different from those of WT plants. *PHYC* was described to act redundantly to *PHYA* in modulating hypocotyl elongation in response to red light [33]. In the LTLT we observed a *PHYC* expression pattern that resembles the pattern found for the *PHYA* gene, while a different expression pattern was observed in the STLT. Despite its well-recognized role in SAS promotion [14], the *PHYB* expression levels showed only minimal changes in response to the different LTLTs. Similarly, Filiault et al. found no correlation between *PHYB* mRNA levels and hypocotyl elongation [34]. However, the *phyB* mutant showed a pronounced L/P decrease in response to the CoeLux® light type, significantly different from that observed in WT plants, suggesting the involvement of this gene in the response to this light type. The *PHYE* gene is known to be closely related to *PHYB*, approximately 55% identity, and was reported to be expressed in the same cell types [35]. In the LTLT it showed an expression pattern resembling that of *PHYB*. The *PHYD* gene is known to act in the SAS by controlling flowering time and leaf area [36], with highly overlapping functions with the *PHYB* gene [35]. However, the expression pattern of the *PHYB* and *PHYD* genes showed to be slightly different, especially in the LTLT, underlying a different regulation at the transcriptional level.

The photomorphogenic responses to UV-B mediated by the UVR8 photoreceptor are well documented [13], however, little is known about the involvement of UVR8 in the perception of light outside the UV-B wavelengths. Despite the absence of UV-B light under the lighting systems used in this study, we observed a change in the expression levels of this gene in response to both light spectrum and light intensity, suggesting the involvement of this protein in other light-dependent mechanisms apart from UV-B perception.

### Genes of different photoreceptors families showed similar expression patterns in the LTLT

In the long-term light treatment, genes of different photoreceptors families showed a similar expression pattern. Among these genes, *PHYB* and *PHOT2* showed no transcriptional changes in response to both light intensity and spectrum. While the second group of genes, composed of *PHYA*, *PHYC*, *PHYD*, *CRY1*, *CRY2*, *PHOT1*, and *UVR8*, showed a peculiar pattern with a marked difference between the two light types applied. Under the HPS light type, the expression levels are raising with the decrease of light intensity, while they remain nearly constant at a high fold under the CoeLux® light type. The higher expression levels of these genes at 120 μmol m^-2^s^-1^ could explain the onset of a more severe SAS under the CoeLux® light type. However, at the lower light intensities, the difference between the two light types is lost, and the expression under the HPS light type is overcoming the expression under the CoeLux® light type, revealing that SAS responses are regulated not only on the transcriptional level of photoreceptors genes. These data suggest that the response to light quality is not independent of light intensity; indeed, the light intensity seems to plays a crucial role in shaping the response of plants to the light spectrum of the CoeLux® light type.

A diverse plethora of photoreceptors is involved in the responses to the CoeLux® light type, including PHYs, CRYs, PHOTs and, probably, also the UVR8 gene. We hypothesized that the molecular signalling activated by the CoeLux® light type could also reflect in a higher expression of CRYs and PHOTs and a lower expression of PHYs. However, our hypothesis was only partially confirmed since CRYs and PHOTs were more expressed only at the higher light intensities, as well as PHYs were less expressed only at the lower light intensities. Furthermore, genes of different families of photoreceptors showed common response patterns, with no marked differences between photoreceptors of different gene families. Since similar response patterns were observed for these genes, the existence of a common upstream regulation of mRNA transcription can be speculated.

### Photoreceptors respond differently to the STLT

In the short-term light treatment, each gene showed a peculiar expression pattern in response to the light turning on. Statistically significant differences between the two light types analysed in our study were observed for the majority of the genes, suggesting that the short-term responses of plants to altered light quality includes also the adjustment of the expression levels of photoreceptors. However, these differences were of brief duration and were observed for no more than two consecutive sampling points, indicating the involvement of other mechanisms that lead to the regulation of the genes of photoreceptors. Studies on the circadian clock regulation, using the luciferase reporter system [37], showed that the genes expression of photoreceptors is subjected to a solid regulation by the circadian clock of the plant and that several days are needed to adapt the expression of these genes to the new light conditions.

## Conclusion

Overall, our expression data fail to fully explain the morpho-physiological differences observed between plants grown under the CoeLux® and the HPS light type, suggesting that the response to light quality and intensity is determined also by the activity of the photoreceptors rather than by their expression level alone. Further studies are needed to integrate the information about light regulation of mRNA profiles with the protein content and functioning of these photoreceptors. Moreover, the study of the expression levels of downstream regulatory factors, like *HY5*, *COP1*, *HFR1*, and PIFs, could provide further knowledge about the responses of plants to the CoeLux® light type and provide a significant starting point for the development of CoeLux-adapted plant strains.

## Supporting information

S1 Data (XLSX)
